# Muramyl dipeptide-mediated immunomodulation on monocyte subsets exerts therapeutic effects in a mouse model of Alzheimer’s disease

**DOI:** 10.1186/s12974-020-01893-3

**Published:** 2020-07-22

**Authors:** Adham Fani Maleki, Giulia Cisbani, Marie-Michèle Plante, Paul Préfontaine, Nataly Laflamme, Jean Gosselin, Serge Rivest

**Affiliations:** 1 Neuroscience Laboratory, CHU de Quebec Research Center and Department of Molecular Medicine, Faculty of Medicine, Laval University, 2705 Laurier Boulevard, Quebec City, QC G1V 4G2 Canada; 2 Laboratory of Innate Immunity, CHU of Quebec Research Center and Department of Molecular Medicine, Faculty of Medicine, Laval University, 2705 Laurier Boulevard, Quebec City, QC G1V 4G2 Canada

**Keywords:** NOD2 receptor, Monocytes, Microglia, Macrophages, Alzheimer’s disease, Immunotherapy, Brain blood vessels, Cerebral amyloid angiopathy, Synapse

## Abstract

**Background:**

Muramyl dipeptide (MDP) is a component derived from minimal peptidoglycan motif from bacteria, and it is a ligand for the NOD2 receptor. Peripheral administration of MDP converts Ly6C^high^ into Ly6C^low^ monocytes. Previously, we have shown that Ly6C^low^ monocytes play crucial roles in the pathology of a mouse model of Alzheimer’s disease (AD). However, medications with mild immunomodulatory effects that solely target specific monocyte subsets, without triggering microglial activation, are rare.

**Methods:**

Three months old APP_swe_/PS1 transgenic male mice and age-matched C57BL/6 J mice were used for high frequency (2 times/week) over 6 months and low frequency (once a week) over 3 months of intraperitoneally MDP (10 mg/kg) administrations. Flow cytometry analysis of monocyte subsets in blood, and behavioral and postmortem analyses were performed.

**Results:**

Memory tests showed mild to a strong improvement in memory function, increased expression levels of postsynaptic density protein 95 (PSD95), and low-density lipoprotein receptor-related protein 1 (LRP1), which are involved in synaptic plasticity and amyloid-beta (Aβ) elimination, respectively. In addition, we found monocyte chemoattractant protein-1(MCP-1) levels significantly increased, whereas intercellular adhesion molecule-1(ICAM-1) significantly decreased, and microglial marker (Iba1) did not change in the treatment group compared to the control. In parallel, we discovered elevated cyclooxygenase-2 (COX2) expression levels in the treated group, which might be a positive factor for synaptic activity.

**Conclusions:**

Our results demonstrate that MDP is beneficial in both the early phase and, to some extent, later phases of the pathology in the mouse model of AD. These data open the way for potential MDP-based medications for AD.

## Background

MDP is derived from minimal bioactive peptidoglycan motif from most Gram-negative and -positive bacteria [1] and mediates its effects on the immune response via NOD2 receptor [1, 2]. This receptor is a member of the NLR family of leucine-rich repeat proteins [1] and is strongly expressed in monocyte precursors that have the ability to differentiate into pro-inflammatory and patrolling subsets and macrophages once infiltrating tissues [3].

In humans, monocyte subsets are characterized by expression levels of CD14 and CD16, as being, classical (CD14^++^CD16^−^), intermediate (CD14^++^CD16^+^), and non-classical (CD14^+^ CD16^++^) subsets [4]. In mice, proinflammatory monocytes are characterized by a combination of surface markers (CX3CR1^low^CCR2^+^Ly6C^high^), whereas patrolling monocytes are defined as CX3CR1^high^CCR2^−^Ly6C^low^ cells [5–9]. Proinflammatory monocytes are involved in inflammatory responses, extravasate in inflamed tissues in a CCR2-dependent manner, and thus contribute to local inflammation. On the other hand, patrolling monocytes (also referred to as anti-inflammatory) establish the resident regulatory patrolling monocyte population [10]. Ly6C^low^ monocytes are resident phagocytes that patrol the lumen of blood vessels and enhance tissue repair [10].

AD is characterized by the chronic activation of innate immune cells within the CNS. AD is associated with Aβ accumulation in the parenchyma and cerebral vasculature due to impaired clearance of the neurotoxic Aβ_1–40_ and Aβ_1–42_ peptides [11, 12]. Several lines of evidence indicate that cerebral amyloid angiopathy (CAA) acts as a significant contributor to the AD pathology [13, 14]. CAA is mainly caused by an impaired Aβ clearance from the cerebral vasculature along perivascular lymphatic drainage pathways. Having more than 90% prevalence in AD patients clearly shows its significant impact on AD pathology and cognitive decline [13, 14].. More importantly, there is a constant equilibrium between Aβ vascular/peripheral and parenchymal levels [15, 16]. Therefore, the clearance of Aβ in perivascular spaces reduces the burden in the parenchyma through equilibrium-driven redistribution [16, 17].

The blood-brain barrier (BBB) structure limits access to select soluble molecules and circulating leukocytes in the CNS [17, 18]. Among leukocytes, monocytes and monocyte-derived perivascular macrophages have a crucial role in AD. Indeed, evidence from previous studies suggests that monocyte-derived perivascular macrophages are highly efficient for Aβ phagocytosis [19, 20]. In parallel, several studies highlighted the crucial effect of Ly6C^low^ monocyte subset in AD. There is a reduction in non-classical CD14^+^CD16^++^ monocytes in AD patients compared with mild cognitive impairment patients or age-matched healthy controls [21]. Our group has shown that Ly6C^low^ monocytes internalize Aβ and efficiently eliminate Aβ microaggregates and transport them from the brain microvasculature to the blood circulation in a two-photon microscopy study [22].

In this study, we investigated whether the immunomodulatory effects of MDP could influence the neuropathology of an APP mouse model of AD. We found that MDP administrations converted Ly6C^high^ into Ly6C^low^ monocytes, which was associated with improvement in memory function together with the increased expression of markers of synaptic plasticity and Aβ clearance.

## Methods

### Animal Care

All protocols were performed according to the Canadian Council on Animal Care guidelines, as administered by the Laval University Animal Welfare Committee. All experiments were approved by the local committee. All efforts were made to avoid their suffering. All mice were maintained in a pure C57BL/6 J background, bred in house, and newborn pups were genotyped with PCR as advised by Jackson Laboratory protocols. All animals were housed up to four per cage in temperature and light-controlled room (12 h light cycles from 7 am to 7 pm) and were fed (mouse chow) and allowed to drink water ad libitum. All mice were monitored for health status, including weight loss throughout all experimental protocols.

### APP model and MDP treatment

APP_Swe_/PS1 expressing the chimeric mouse/human amyloid precursor protein (Mo/HuAPP695swe) and a mutant human presenilin 1 (PS1-dE9) under the control of independent mouse prion promoter elements [B6.CgTg(APPswe,PSEN1dE9)85Dbo/J]. A total of fifty-five 3 months old male APPswe/PS1 transgenic mice and twenty-five age-matched C57BL/6 J wild-type mice (WT) were utilized. Mice were injected one/two times per week with either MDP commercially available (Catalog # tlrl-MDP, Version # 16A22-MM, Invivo Gen) diluted in saline (10 mg/kg) or vehicle (saline 0.9%).

### Flow cytometry

Blood samples were collected from the submandibular vein and kept in EDTA coated vials on a rotator for < 1 h, and fluorescence-activated cell sorting (FACS) analysis was performed as described by [23, 24]. FACS and data acquisition were performed using SORP LSR II and FACSDiva softwares (both from BD), respectively. Results were analyzed with the FlowJo software (v10.0.7). For details on flow cytometry protocol, see Additional file 1, Fig. 2 [24].

### Sacrifices

All mice were sacrificed via intracardiac perfusion with 0.9% saline, followed by 4% PFA pH 7.4. The brains were then retrieved, post-fixed 10–24 h in 4% PFA pH 7.4, and transferred in 4% PFA pH 7.4 + 20% sucrose for a minimum of 15 h. In another set of experiments, brains were retrieved, and one hemisphere was snap-freeze for protein extraction while the other hemisphere was fixed in 4% PFA pH 7.4 + 20% sucrose. Brains were sliced in coronal sections of 25-μm thickness with a freezing microtome (Leica Microsystems), serially collected in an anti-freeze solution and kept at − 20 °C until usage.

### Post-mortem analysis

#### Immunofluorescence

Brain sections were washed four times for 5 min in KPBS and then blocked in KPBS containing 1% BSA, 4% NGS, and 0.4% Triton X-100. The tissues were incubated overnight at 4 ^°^C with the primary Iba-1 antibody (1: 2,000; Wako Chemicals) and monoclonal anti-Aβ (6E10, 1: 3000; Covance). After washing four times for 5 min in KPBS, tissues were incubated in the appropriate secondary antibody (IgG anti-mouse Alexa 488; Thermofisher and IgG anti-rabbit CY3; Jackson Immunoresearch) for 2 h at room temperature. Following further washes in KPBS and incubation with DAPI, the sections were mounted onto Micro Slides Superfrost Plus glass slides and coverslipped with Fluoromount-G (Electron Microscopy Sciences).

#### Image acquisition and analyses

Image acquisition of fluorescent staining images was performed using a Zeiss LSM800 confocal microscope supported by the Zen software (2.3 system) using the × 4 and × 40 lenses, as described previously [25]. The number of 6E10, Iba-1 associated with plaques, was quantified by unbiased stereological analysis [26] using the Stereo Investigator software (version 6.02.1, MicroBrightfield) attached to a Nikon C80i microscope equipped with a motorized stage (Ludl) attached to Microfire CCD color camera (Optronics). Four to six sections were analyzed for each animal.

#### Soluble Aβ_1–42_/Aβ_1–40_ ELISA

Brain levels of soluble Aβ_1–42_ and Aβ_1–40_ were quantified by using the Human Amyloid β42 and Human Amyloid β40 Brain ELISA kits (Millipore, Billerica, MA, USA). The experimental procedure was performed according to the manufacturer’s instructions [27].

#### Western blot analysis

Hippocampus and cortex brain proteins were lysate, as previously described [27]. Proteins were then loaded in 4–15% agarose precast gels (Biorad) and electroblotted onto 0.45 μm Immobilon PVDF membranes. Membranes were immunoblotted with various primary antibodies, as described in Table 1, followed by the appropriate horseradish peroxidase (HRP)-conjugated secondary antibodies and revealed by enhanced chemiluminescence plus (ECL) solution (GE Healthcare Life Sciences). Quantification was done by determining the integrative density of the bands using the Thermo Scientific Pierce myImage Analysis Software v2.0. Optical values were normalized over actin.

**Table 1:** Represents antibodies used for immunoblotting analyses

Antibody	Company	Molecular weight	Species	Dilution	Secondary antibody dilution	Notes
Actin	Millipore	42 kDa	Mouse	1/50,000	1/20000	
APP	Millipore	≈ 100 kDa	Mouse	1/2000	1/5000	antigen retrival (Michaud, Hallé et al. 2013)
Cox-2	Santa Cruz	≈ 75 kDa	Goat	1/1000	1/5000	
Iba-1	Wako	18 kDa	Rabbit	1/1000	1/5000	
ICAM	Santa Cruz	85–110 kDa	Goat	1/500	1/1000	
LRP1	CEDARLANE	85 kDa	Rabbit	1/1 000	1/40,000	
MCP1	Cell Signaling	13 kDa	Rabbit	1/1 000	1/5000	
PSD95	Neuromab	95 kDa	Mouse	1/2 000	1/20,000	
Synaptophysin	Thermo Fisher Scientific	34 kDa	Mouse	1/10 000	1/100,000	
Trem2	R&D Systems	40 kDa	Rabbit	1/500	1/2000	antigen retrival (Michaud, Hallé et al. 2013)
VCAM	Santa Cruz	90–100 kDa	Rabbit	1/1 000	1/10,000	
NFkB p50	CEDARLANE	50 kDa	Rabbit	1/1000	1/10,000	

Table 1 represents antibodies used for immunoblotting analyses and all related information, including the name of the company, molecular weight, species, secondary antibodies, and dilution rates

### Behavioral tests

#### Open field

The open field was performed to evaluate anxiety-like behaviors, exploration habits, and also locomotor activity, as described by Hui et al. [28]. Each mouse was individually recorded and analyzed by the ANY-maze system.

#### Water T maze

The water T maze assay was performed, according to Guariglia et al. [29]. The pool was filled with 23^°^ C (± 1 C) water to a depth of 13 cm, which was 1 cm above the surface of the platform. Mice were trained to swim to a particular arm of the T maze and to remain on a submerged platform for 5 s. Mice had to complete six out of eight trials without error for two consecutive days out of three days to reach the learning criterion. The same criterion was considered for the reversal phase.

### Statistics

Data are expressed as the mean ± SEM. A comparison between two groups was conducted using post hoc unpaired *t* tests. Comparisons between more than two treatment groups were performed using either one-way analysis of variance (ANOVA) or two-way repeated-measures ANOVA, followed by Tukey’s post-hoc test or uncorrected Fisher’s Least Significant Difference test. Values were statistically significant if *p* < 0.05. All analyses were performed using GraphPad Prism Version 6 for Windows (GraphPad Software, San Diego, CA, USA) and SAS 9.4 (SAS Institute Inc., Cary, NC, USA). All panels were assembled using Adobe Photoshop CS5 (version 12.0.4) and Adobe Illustrator CS5 (version 15.0.2).

## Results

### Chronic and high frequency of MDP administration in the APP mouse model of AD show a slight improvement in memory function

**Fig. 1:**
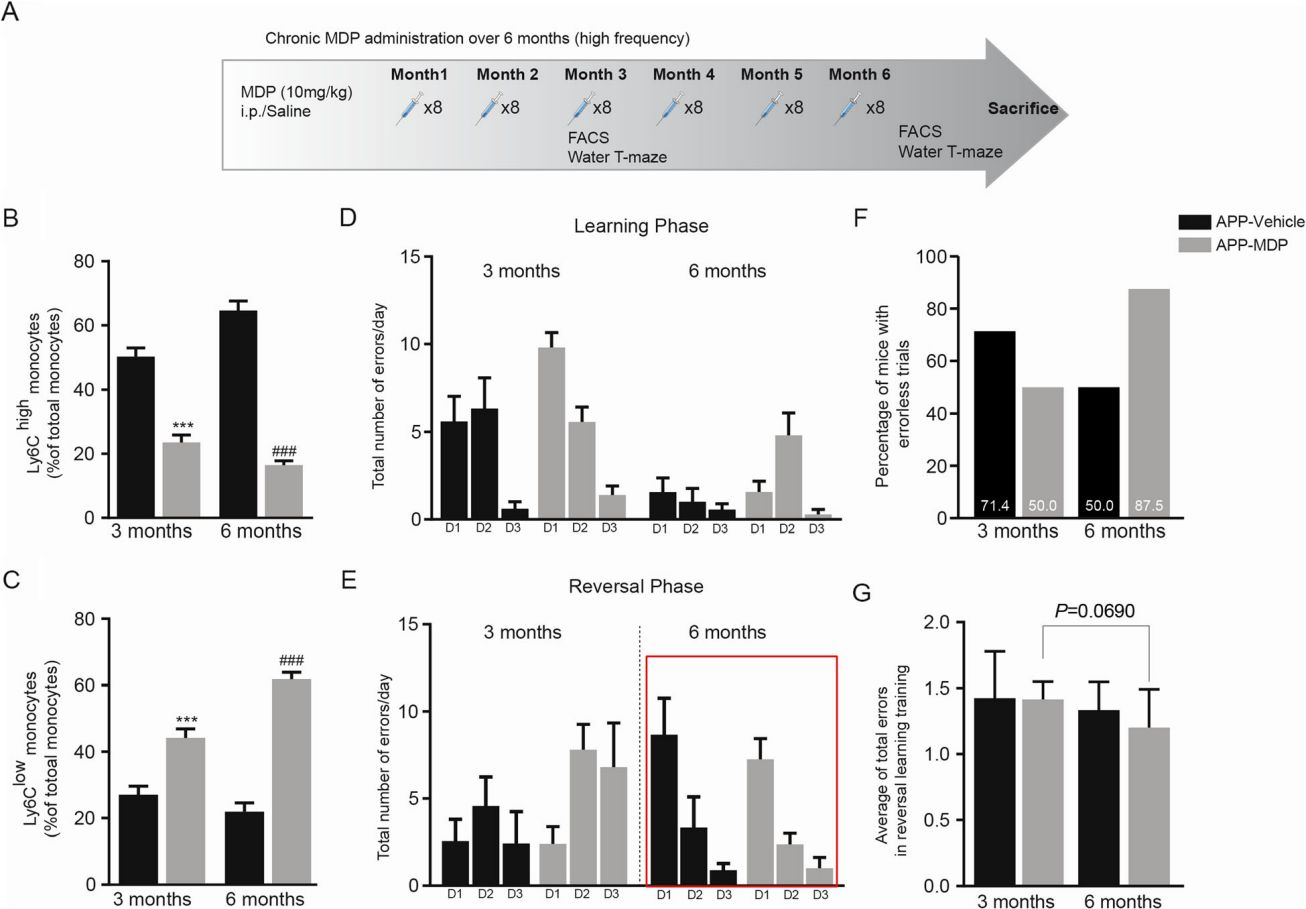
Regulation of monocyte subsets and a slight improvement in memory function following chronic MDP administration over 6 months (high frequency) in APP mice. **a** Representative timeline of chronic MDP administration over 6 months (high frequency) in APP mice, *n* = 10 mice per group. **b** Percentage of blood inflammatory Ly6C^hi^ monocytes at two time points (3 and 6 months) following chronic MDP administration over 6 months (high frequency) in APP mice. Data are expressed as the means ± SEM; ^***^P < or = 0.0004 vs. APP vehicle in 3 months, ^###^*P* < or = 0.0004 vs. APP vehicle in 6 months. **c** Percentage of blood Ly6C^low^ patrolling monocytes at two time points (3 and 6 months) following chronic MDP administration over 6 months (high frequency) in APP mice. Data are expressed as the means ± SEM; ^***^P < or = 0.0004 vs. APP-MDP in 3 months, ^###^*P* < or = 0.0004 vs. APP-MDP in 6 months. **d** The total number of errors made on day 1 (D1), day 2 (D2), and day 3 (D3) in APP-MDP and APP-vehicle groups in learning performance in position habit acquisition at the two time points (3 and 6 months). Data analyzed using two-way ANOVA for the time points and treatments. **e** The total number of errors made on day 1 (D1), day 2 (D2), and day 3 (D3) in APP-MDP and APP-vehicle groups in learning performance in reversal learning training at the two time points (3 and 6 months). Data analyzed using two-way ANOVA for the time points and treatments. **f** Percentage of mice in APP-MDP and APP-vehicle groups made errorless trials in day 1 in reversal learning training at the two time points (3 and 6 months). **g** Average of total errors in APP-MDP and APP-vehicle groups in learning performance in reversal learning training at the two time points (3 and 6 months). Data analyzed using two-way ANOVA for the time points and treatments

Our team has previously demonstrated a critical role of Ly6C^low^ monocytes in Aβ clearance within the cerebrovascular system. Indeed, Ly6C^low^ monocytes are able to associate within Aβ-positive veins but not arteries, internalize Aβ, and efficiently eliminate Aβ microaggregates and transport them from the brain microvasculature to the blood circulation [22]. Immune modulation mediated by MDP in shifting monocyte subsets towards Ly6C^low^ prompted us to assess potential therapeutic effects of MDP in APP mice. We chronically administered MDP twice a week (high frequency) in 3-month-old APP mice over 6 months (Fig. 1a). We then evaluated the circulating monocyte subsets at both 3 and 6 months following the beginning of the injections. The APP mice develop an Alzheimer-like phenotype at 6 months of age. In parallel, 4–6 months old APP mice develop small and punctate Aβ aggregates accumulation on specific blood vessels [22]. Thus, these time points were chosen to evaluate whether MDP is capable of delaying disease onset (3 months following the first MDP injection) and maintain the phenotype over time (6 months after the first MDP injection). The drug was able to modulate Ly6C^high^ monocytes towards the Ly6C^low^ subset at both 3- and 6-month post-injection times (Fig. 1b, c). To assess whether MDP affects cognitive behavior, we performed the water T-maze test. We observed that APP mice that received MDP did not significantly differ from their counterparts that received saline both during the learning and reversal phases of the water T-maze test (Fig. 1d, e). Nevertheless, shifting in the percentage of mice with errorless trials in both groups at the two time points suggests a slight improvement in performing the memory test in the treatment group. Indeed, at 3 months post-injection, the percentages of mice with errorless trials were 71.4 and 50 in control and treatment groups, respectively. On the contrary, at 6 months post-injection, we observed that the control group showed a reduction in the percentage and reached 50%. In contrast, the treatment group demonstrated an increase in the percentage (87.5%) of mice with errorless trials (Fig. 1f). Moreover, analysis of the average of total errors in the reversal phase of the test between treatment and control groups at the two time points also suggests a slight improvement in memory function. More precisely, we did not find a significant change in the average of total errors between treatment and control groups in the two time points. Nonetheless, the treatment group (but not the control group) showed a tendency (*p* = 0.0690) to have a lower number of total errors at the second time point when compared with the first time point (Fig. 1g). In the open field test, the results did not show any significant difference between the group treated with saline or MDP, indicating that the treatment caused neither anxiety-like behaviors nor locomotor activity problems (data not shown). Overall, these results suggest that monocytes are modulated in APP mice, and MDP treatments slightly improved cognitive deficits of the mice when the disease is established.

### A chronic and low frequency of MDP administration improves cognitive deficits in the APP mouse model of AD

**Fig. 2:**
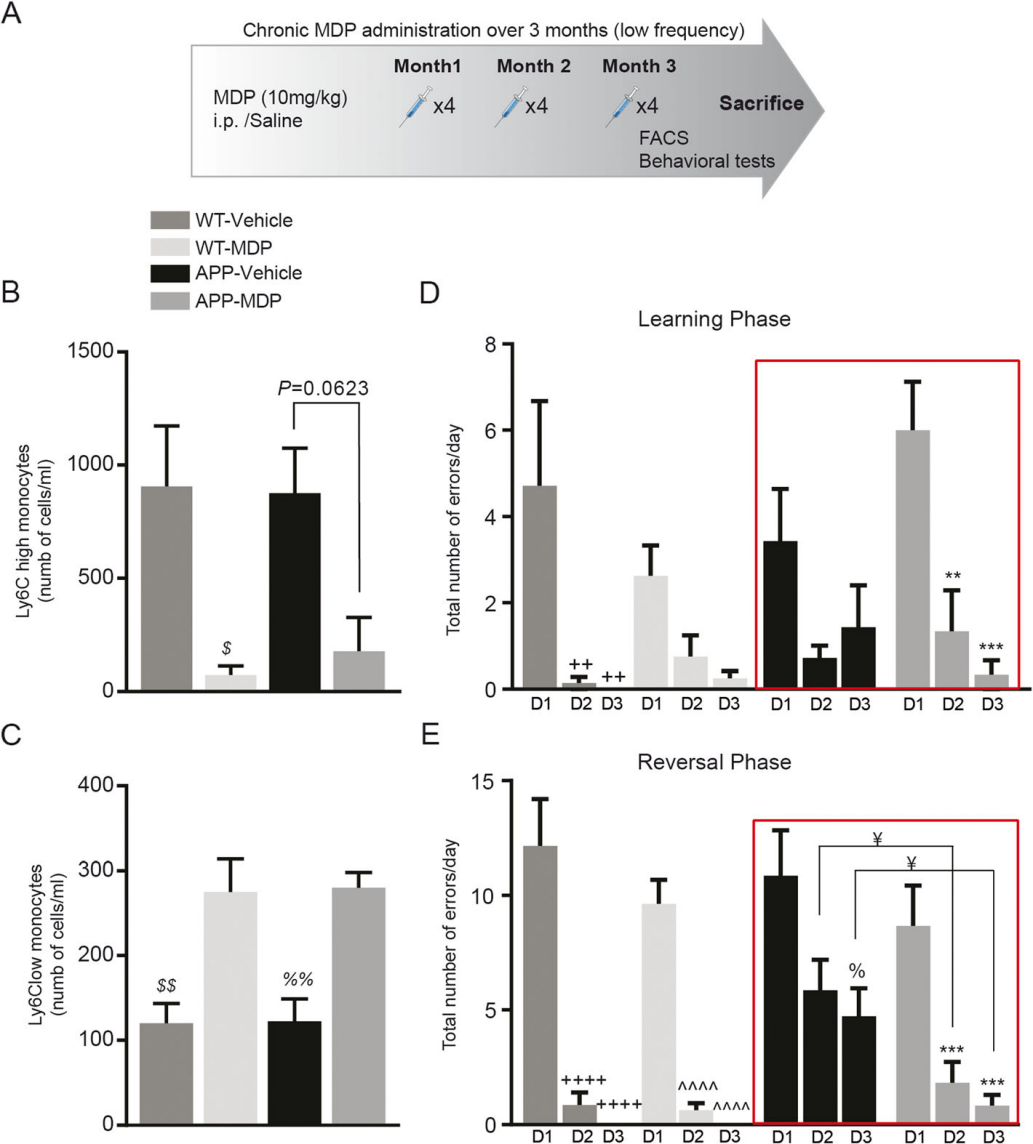
Regulation of monocyte subsets and improvement in memory deficits following chronic MDP administration over 3 months (low frequency) in APP mice. **a** Representative timeline of chronic MDP administration over 3 months (low frequency) in APP and WT mice, APP *n* = 10 mice per group, and WT *n* = 5 mice per group. **b**, **c** Absolute count of blood inflammatory Ly6C^hi^ monocytes in WT and APP mice and following chronic MDP administration over 3 months (low frequency). Data are expressed as the means ± SEM; ^$^*P* < or = 0.01 vs. WT-vehicle. **c** Absolute count of blood Ly6C^low^ monocytes in WT and APP mice and following chronic MDP administration over 3 months (low frequency). Data are expressed as the means ± SEM; ^$$^*P* < or = 0.003 vs. WT-MDP, ^%%^*P* < or = 0.007 vs APP-MDP. **d** The total number of errors made on day 1 (D1), day 2 (D2), and day 3 (D3) in WT and APP mice in learning performance in position habit acquisition following chronic MDP administration over 3 months (low frequency). Data are expressed as the means ± SEM; ^++^*P* < or = 0.002 vs. WT-vehicle D1, ^**^P < or = 0.003 vs. APP-MDP D1, ^***^P < or = 0.0004 vs APP-MDP D1. Data analyzed using two-way ANOVA for the time points and treatments. **e** The total number of errors made on day 1 (D1), day 2 (D2), and day 3 (D3) in WT and APP mice in reversal performance in position habit acquisition following chronic MDP administration over 3 months (low frequency). Data are expressed as the means ± SEM; ^¥^*P* < or = 0.0266 vs. APP-vehicle D2 and D3, ^++++^*P* < or = 0.0001 vs. WT-vehicle D1, ^^^^^^*P* < or = 0.0001 vs. WT-MDP D1, ^%^*P* < or = 0.0092 vs. APP-vehicle D1, ^***^P < or = 0.0008 vs. APP-MDP D1. Data analyzed using two-way ANOVA for the time points and treatments

We then tested whether chronic MDP injection in APP and WT mice is at a lower frequency (once a week) over 3 months (Fig. 2a). Similar to the high-frequency MDP administration, a low-frequency MDP administration was able to shift monocyte subsets towards the Ly6C^low^ phenotype (Fig. 2b, c). In the water T-maze test, first, we analyzed learning curves in the training phase using a two factor (treatment × day) ANOVA with repeated measures on day. We only observed a significant effect of day between groups (*F* (2, 48) = 21.18, *P* < 0.0001). Although we observed a significant effect of day between groups, Post-hoc comparisons showed no difference for every single day when we compared all groups (even WT-vehicle versus APP-vehicle). That might be related—at least partially—to the fact that the water T-maze has a single choice point with only two alternatives, which increases the possibilities of success (by default, the probability of choosing the correct arm is 50%) reviewed in details by Sharma and colleagues [30]. Therefore, we performed a repeated-measures ANOVA test on intra-group performance to see whether every group during 3 days training phase was able to make statistically significantly fewer errors. Although at different rates, WT groups overall showed improvement by making fewer errors on the second and third days in comparison with the first day of the training phase (Fig. 2d). Moreover, the improvement in the WT-vehicle group reached statistical significance (Fig. 2d), whereas mice in the APP control group did not show any significant difference on the same days. More importantly, they made more errors on the third day compared with the second day, indicating that they were impaired in their ability to consolidate the entering right arm following two consecutive days of training (Fig. 2d). In contrast, the APP treatment group made significantly fewer errors in the second and third days in comparison with the first day of training phase (Fig. 2d).

Next, using a two factor (treatment × day) ANOVA with repeated measures on day, we analyzed all groups in the reversal phase, which is the most important phase of the water T-maze test. ANOVA revealed a significant effect of treatment (*F* (2, 48) = 73.12, *P* < 0.0001) and a significant effect of day (*F* (3, 24) = 6.006, *P* = 0.0033). Post-hoc comparisons (uncorrected Fisher’s Least Significant Difference test) revealed significant differences between the control groups and the treatment group. More precisely, on the second and third days, there was a significant difference between APP control and treatment groups (Fig. 2e). Although it is not shown in the figure, we found a significant difference on the second day, in both WT groups versus APP control (*P* < 0.0016), and on the third day, in both WT groups versus APP control (*P* < 0.0042). We also reported the results from repeated-measures ANOVA on intra-group tests for the reversal phase (Fig. 2e).

Besides the water T-maze test, we observed the same results as the high-frequency protocol from the open field test (data not shown). These results suggest that chronic administration of MDP at lower frequency is sufficient to delay the appearance of an Alzheimer-like phenotype. Collectively, the behavioral test results obtained from both protocols suggest that low-frequency MDP administration (once per week) is more effective than high-frequency administration (twice per week).

### MDP-derived memory improvement is not dependent on the change in Aβ levels and microglial activation

Microglial cells play a key role in AD pathogenesis by regulating Aβ levels in the brain via uptake and degradation processes. Therefore, we evaluated whether MDP treatment could impact Aβ accumulation and microglial functions. We measured the number of Iba1-positive microglia associated with 6E10-positive plaques as well as the number of immunostained plaques in both the hippocampus and cortex of APP mice that received MDP or saline. We did not observe any significant difference between the two groups (see Additional file 1, figure S1A- S1H). We subsequently assayed soluble Aβ40 and Aβ42 levels in cortex and hippocampus by specific ELISA immunoassays, and even in this case, the results showed no significant difference between treatment and control groups (see Additional file 1, figure S1I and S1J). We next measured several markers in both protocols. To avoid redundancy, in this section, as well as the following sections, we have presented the results only from the high-frequency protocol.

**Fig. 3:**
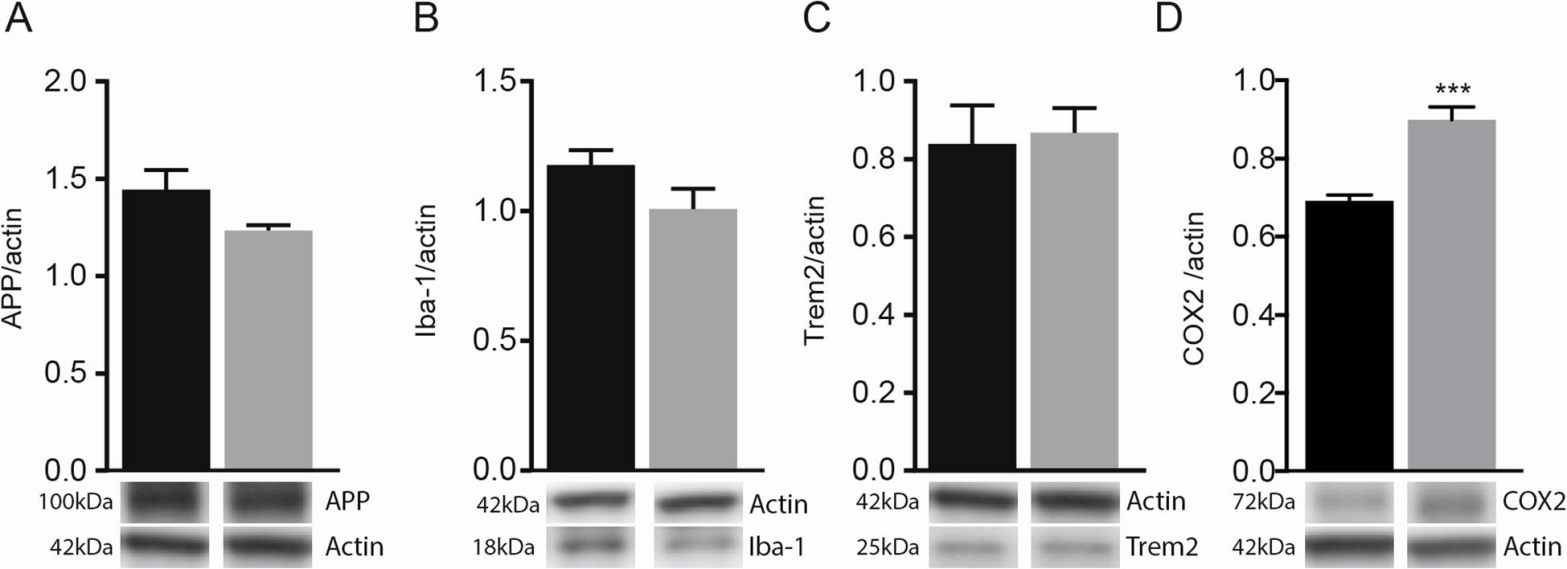
MDP treatment had no effect on microglial activation, and APP levels, but upregulated COX2 levels in APP mice. **a** Immunoblot analysis of APP levels in the cortex and hippocampus of APP mice treated with vehicle and MDP. **b**, **c** Immunoblot analysis of Iba1 and TREM2 levels in the cortex and hippocampus of APP mice treated with vehicle and MDP. **d** Immunoblot analysis of COX2 levels in the cortex and hippocampus of APP mice treated with vehicle and MDP. Data are expressed as the means ± SEM; ^***^P < 0.0001 vs. APP-MDP

As Aβ is produced through sequential cleavage of APP, catalyzed by β- and γ-secretase [31], we measured the expression level APP by immunoblot, and we did not observe any change in APP level in both groups, (Fig. 3a). In addition, we determined the expression levels of Iba1, TREM2, and nuclear factor-kB (NF-kB, P50) in the hippocampus of APP mice treated with MDP or saline, and no differences were observed (Fig. 3b, c). Finally, we observed significantly higher levels of COX2 in the brain of MDP-treated mice (Fig. 3d), which may be indicative of synaptic plasticity [32–34]. Altogether, these results indicate that the memory/learning improvements observed in behavioral tests are not dependent on the Aβ burden or microglial activation, suggesting other factor(s) involved in MDP-mediated cognitive improvement.

### MDP-derived memory improvement mediated by modification of synaptic function and vascular clearance of Aβ

**Fig. 4:**
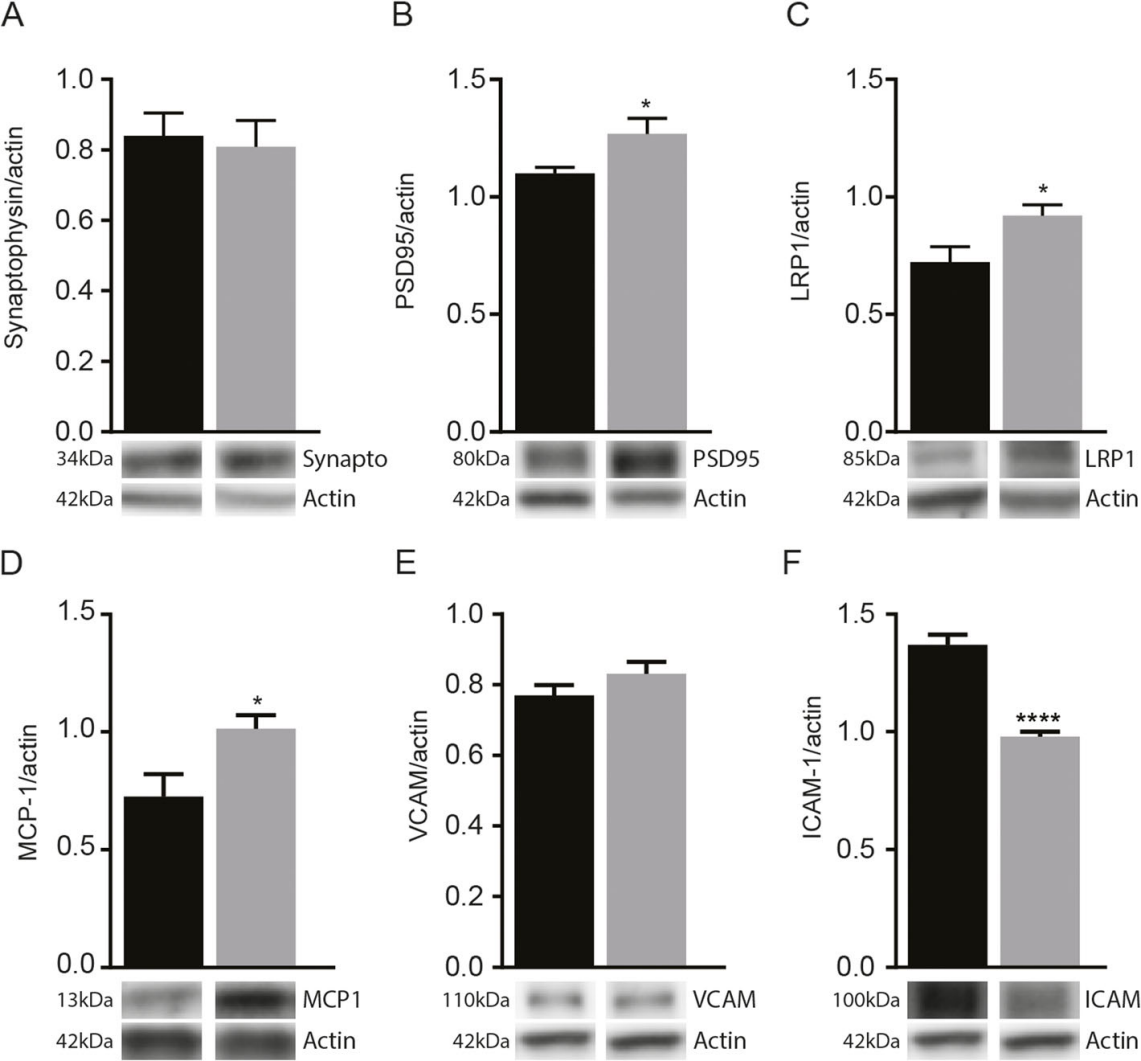
Effect of MDP treatment on key proteins involved in synaptic functions, Aβ vascular clearance, and cerebrovascular monocyte adhesion. **a**, **b** Immunoblot analysis of synaptophysin and PSD95 protein levels, respectively, in the cortex and hippocampus of APP mice treated with vehicle and MDP, *n* = 10 mice per group. Data are expressed as the means ± SEM; ^*^P < or = 0.03. **c**, **d** Immunoblot analysis of LRP1 and MCP-1 protein levels respectively in the cortex and hippocampus of APP mice treated with vehicle and MDP, *n* = 10 mice per group. Data are expressed as the means ± SEM; ^*^P < or = 0.03. **e**, **f** Immunoblot analysis of VCAM and ICAM-1 protein levels, respectively, in the cortex and hippocampus of APP mice treated with vehicle and MDP, *n* = 10 mice per group. Data are expressed as the means ± SEM; ^****^P < or = 0.0001

We next asked if MDP-derived memory improvement is dependent on improvement in synapse formation. Hence, we quantified pre- and postsynaptic puncta (synaptophysin and PSD95) in treatment and control groups. Immunoblot analysis of synaptophysin showed no significant difference (Fig. 4a). However, we found that PSD95 levels significantly increased in APP-MDP compared to those of control mice (Fig. 4b). Consequently, we assessed the LRP1 level as this protein interacts and co-localizes with PSD95 for synapse formation and is a key player to eliminate Aβ across the BBB [35]. Interestingly, LRP1 protein expression levels also increased significantly in the group treated with MDP (Fig. 4c). Altogether, these results indicate that memory improvement mediated by MDP may depend on the enhancement of synaptic plasticity and vascular Aβ clearance.

### Effect of MDP on key proteins involved in cerebrovascular monocyte adhesion

Because we did not observe any differences at the microglial level, we evaluated whether proteins normally associated with monocyte recruitment and vascular adhesion which were modulated following MDP treatments. Monocyte chemoattractant protein-1 (MCP-1) levels play a crucial role in the recruitment monocytes along with the cerebrovascular elements, and they increased significantly in the brain of mice treated with MDP (Fig. 4d). Interestingly, as mentioned before, no significant change was detected for NF-kB (data not shown). We then evaluated the expression levels of vascular cell adhesion molecule-1 (VCAM-1) and intercellular adhesion molecule–1 (ICAM-1). VCAM-1 showed no significant changes, whereas a reduction in the expression level of ICAM-1 was observed in mice treated with MDP when compared to the control group (Fig. 4e, f). Consistent with our previous observations, these results indicate that MDP has no apparent neuroinflammatory effects in the brain but can modulate the levels of chemotactic factors. Finally, the synaptic markers, LRP1, the monocyte recruitment, and vascular adhesion markers, did not show a significant change in WT-MDP when compared with WT-vehicle (data not shown).

## Discussion

Previously, we have shown a critical role of Ly6C^low^ monocytes in the cerebrovascular Aβ clearance by internalization of Aβ and efficiently eliminate Aβ microaggregates from the brain into circulation [22]. Consequently, we examined the potential therapeutic effect of MDP in the APP mouse model of AD. In the first protocol (high frequency), behavioral test results show slight improvement (tendency) in memory function, whereas APP mice treated with MDP in the second protocol (low frequency) demonstrated significant improvement in the memory test. The memory test results obtained from both protocols suggest that the chronic administration of MDP at lower frequency is sufficient to delay the appearance of an Alzheimer-like phenotype. Whether other factor(s) in the two protocols besides the treatment frequency contributed to the difference in memory test results is an open question. In postmortem analysis, we first examined microglial activation and Aβ levels. Nevertheless, we did not observe any change in Aβ burden or microglial activation, suggesting that overall memory improvements observed in behavioral tests, especially low-frequency protocol, are dependent on other factor(s) involved in MDP-mediated cognitive improvement.

Previous reports demonstrated that the degree of synapse loss is a stronger correlate of cognitive decline in AD than counts and/or size of plaques [36–38]. We found that PSD95 protein expression level significantly increased in the APP mice treated with MDP compared to that of control. PSD95 is the most abundant protein in the excitatory postsynaptic density. Furthermore, PSD95 is a master regulator of neuronal plasticity and memory [39] and has previously been shown to be decreased in the APP mouse model of AD [40]. Interestingly, other studies demonstrated the role of PSD95 in interacting and regulating adhesion molecules, signaling proteins, scaffolding proteins, and cytoskeletal proteins [41, 42]. It is then possible that MDP-mediated PSD95 regulation is beyond its role in stabilizing the neuronal circuitry.

**Fig. 5:**
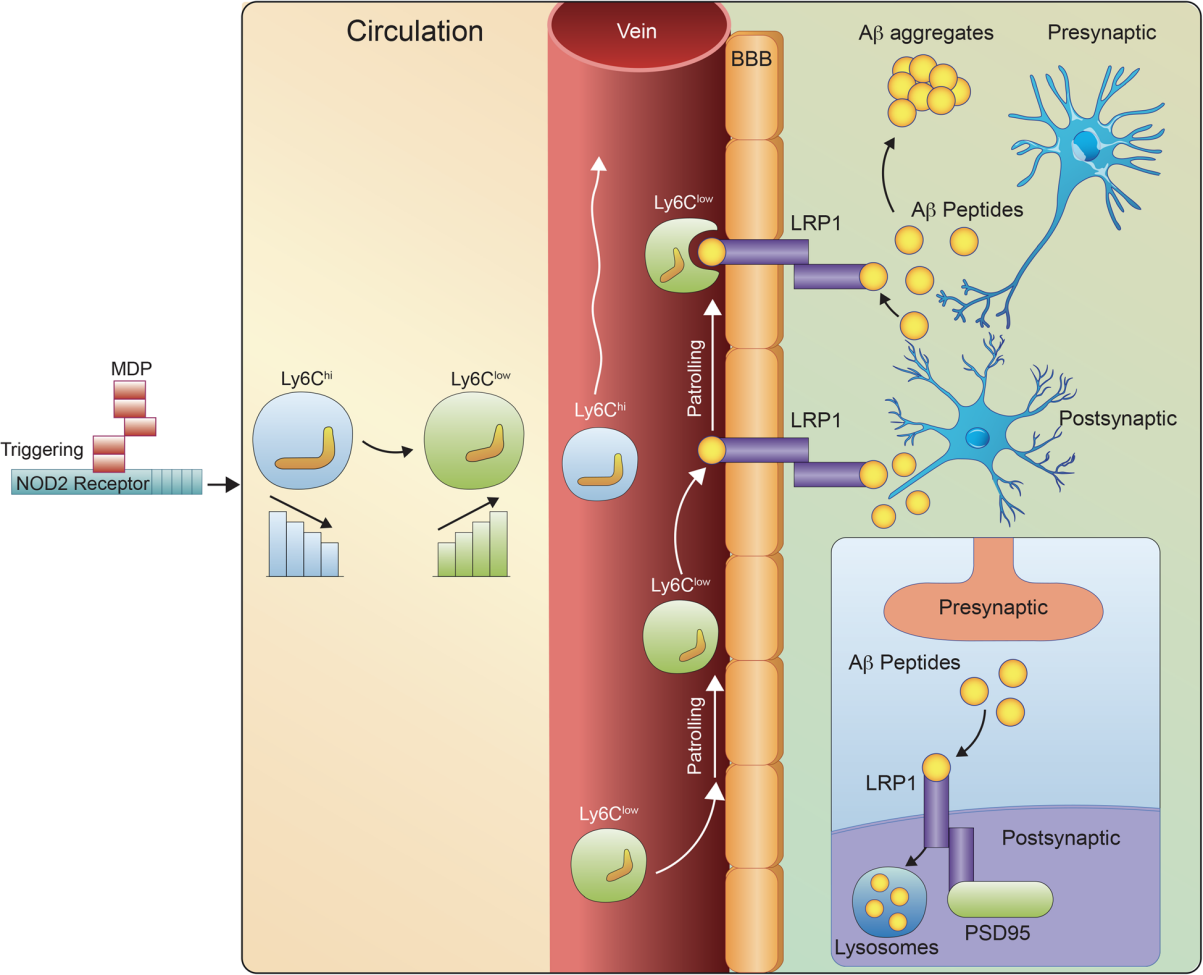
A scheme summarizing MDP-mediated immunomodulatory effects on the APP mouse model of AD via converting inflammatory monocytes into patrolling monocytes. MDP treatments improved cognitive declines and Aβ clearance in APP mice and increased expression levels of PSD95 and LRP1 receptors

PSD95 also interacts and co-localizes with LRP1 [43, 44]. Interestingly, the LRP1 protein expression levels also increased significantly in the group treated with MDP. Accumulating evidence also suggests that LRP1 is a key player in AD pathology at the BBB level [45]. Indeed, LRP1 is involved not only in Aβ endocytosis and cerebral degradation mediated by neurons, but it is also a key player to eliminate Aβ across the BBB [35, 46, 47]. In parallel, we observed a significant increase in COX2 expression levels in the MDP-treated group. While excessive COX2 expression plays a key role in neuroinflammation [48], several studies clearly showed that both constitutive and inducible COX2 play an important role in the refinement of synaptic activity [32, 49]. More importantly, the involvement of COX2 in long-term synaptic plasticity and cognition has been supported by several behavioral tests, reviewed by [50]. Similar to previous reports, we did not observe a significant increase in inflammatory marker expression levels in the brain, but we found a modulation of COX2 expression levels. In the context of MDP treatment, future studies are needed to confirm the positive effect of COX2 on the synaptic function that has been reported previously. Taken together, considering these results, it becomes tempting to suggest that PSD95 and LRP1 are two key factors involved in MDP-derived memory improvement via enhancement of synapse function and vascular Aβ clearance. In addition to the role of LRP1 in vascular Aβ clearance and considering the upregulation of PSD95, it is tempting to propose a role of LRP1 as an endocytic receptor for the neuronal clearance of Aβ. This needs to be further investigated to understand the differential roles of LRP1 in Aβ clearance in the context of MDP mediating immune regulation (Fig. 5).

To further confirm the contribution of peripheral monocyte recruitments in vascular Aβ clearance, we assessed MCP-1 expression levels. Previous reports from our group demonstrated MCP-1-mediated monocyte recruitment in the brain vascular system [51]. MCP-1 protein expression levels increased significantly in APP mice treated with MDP compared to controls. Interestingly, we found no significant change in the protein expression level of the NF-kB. Since NF-kB is an inflammatory mediator involved in MCP-1 production, we believe the increase in MCP-1 expression level may not be dependent on the pro-inflammatory response. To further explore this phenomenon, we next analyzed the endothelial inflammatory biomarkers, VCAM-1, and ICAM-1 (Chakraborty, De Wit, et al. 2017). While VACM-1 showed no significant changes, we observed a significant reduction in ICAM-1 protein expression level in APP mice treated with MDP compared to controls. Consistent with our previous observations, these results indicate that MDP treatments favor chemotactic gradients to allow the recruitment of monocytes/macrophages to the brain vascular system without being associated with neuroinflammation.

Using live intravital two-photon microscopy, we investigated whether MDP-mediated shifting towards Ly6C^low^ monocytes could drive vascular Aβ clearance via Aβ uptake by Ly6C^low^ monocytes (data not shown). We must confirm the data using more mice per group (we have used 1 mouse/group). Nevertheless, based on our preliminary data, crawling GFP^+^ cells in APP_swe_/PS1^+/−^/Cx3CR1^GFP/+^ mouse appeared to be more frequent in blood vessels containing small Aβ aggregates when treated with MDP compared with the vehicle one. Interestingly, following MDP treatment in APP and WT mice, the GFP^+^ cells seem to be more frequent in the APP mouse with blood vessels containing small Aβ aggregates, but not in the WT mouse (data not shown).

This study also raises questions about the effect of MDP on microglial activation and function. We identified no difference between Iba1 protein levels, suggesting that MDP might regulate only systemic myeloid cells, mainly Ly6C^high^ and Ly6C^low^ monocytes. However, this does not rule out an indirect effect of MDP on the activity of microglia. Future studies using different time points after MDP administrations in different disease stages are needed to determine possible indirect effects of MDP, specifically on microglia.

## Conclusions

Our findings demonstrate selective immunomodulatory effects of MDP on a mouse model of AD. Medications that solely target specific monocyte subsets and monocyte-derived macrophages with mild immunomodulatory effects in neurodegenerative disease, without triggering microglial activation, are rare. Here, we have shown the therapeutic effects of MDP administration in an APP mouse model of AD. Furthermore, we have provided solid evidence indicating the potential of MDP in terms of maintaining its therapeutic effect via regulating monocyte subsets in long-term administration (both in WT and APP model). Taken together, our results suggest that MDP may be beneficial in the early phase and, to some extent, late phase of AD.

## Supplementary information

**Additional file 1.** Microglial activation and Aβ burden analysis. Flow cytometry protocols. Details of microglial activation and Aβ load analysis, and flow cytometry protocols for extracellular staining as well as gating strategy

## Data Availability

The data supporting the findings of this study are available from the corresponding author upon request.

## Abbreviations

AD: Alzheimer’s disease
APP: Amyloid precursor protein
Aβ: Amyloid-beta
BSA: Bovine serum albumin
CAA: Cerebral amyloid angiopathy
COX2: Cyclooxygenase-II
DAB: 3,3′-Diaminobenzidine
DAPI: 4′,6-diamidino-2-phenylindole
EDTA: Ethylene diamine tetraacetic acid
ICAM: Intercellular Adhesion Molecule
KPBS: Phosphate-buffered saline containing potassium
MCP-1: monocyte chemoattractant protein-1
MDP: Muramyl dipeptide
NGS: Normal goat serum
PS1: Presenilin 1
TREM2: Triggering receptor expressed on myeloid cells 2
VCAM: Vascular cell adhesion protein
WT: Wild type
