# Supplementary material for: Muramyl dipeptide-mediated immunomodulation on monocyte subsets exerts therapeutic effects in a mouse model of Alzheimer’s disease
Source: J Neuroinflammation. 2020 Jul 22;17:218. doi: 10.1186/s12974-020-01893-3 (PMC7376735; doi:10.1186/s12974-020-01893-3)
Supplement: Supplementary file 1 — Additional file 1. Microglial activation and Aβ burden analysis. Flow cytometry protocols. Details of microglial activation and Aβ load analysis, and flow cytometry protocols for extracellular staining as well as gating strategy [file 12974_2020_1893_MOESM1_ESM.docx]

# A. Microglial activation and Aβ burden analysis.

**A1. Stereology and ELISA analysis showed MDP had no effect on microglial activation and Aβ burden in both high frequency (figure S1) and low frequency (data not shown) protocols.**

**
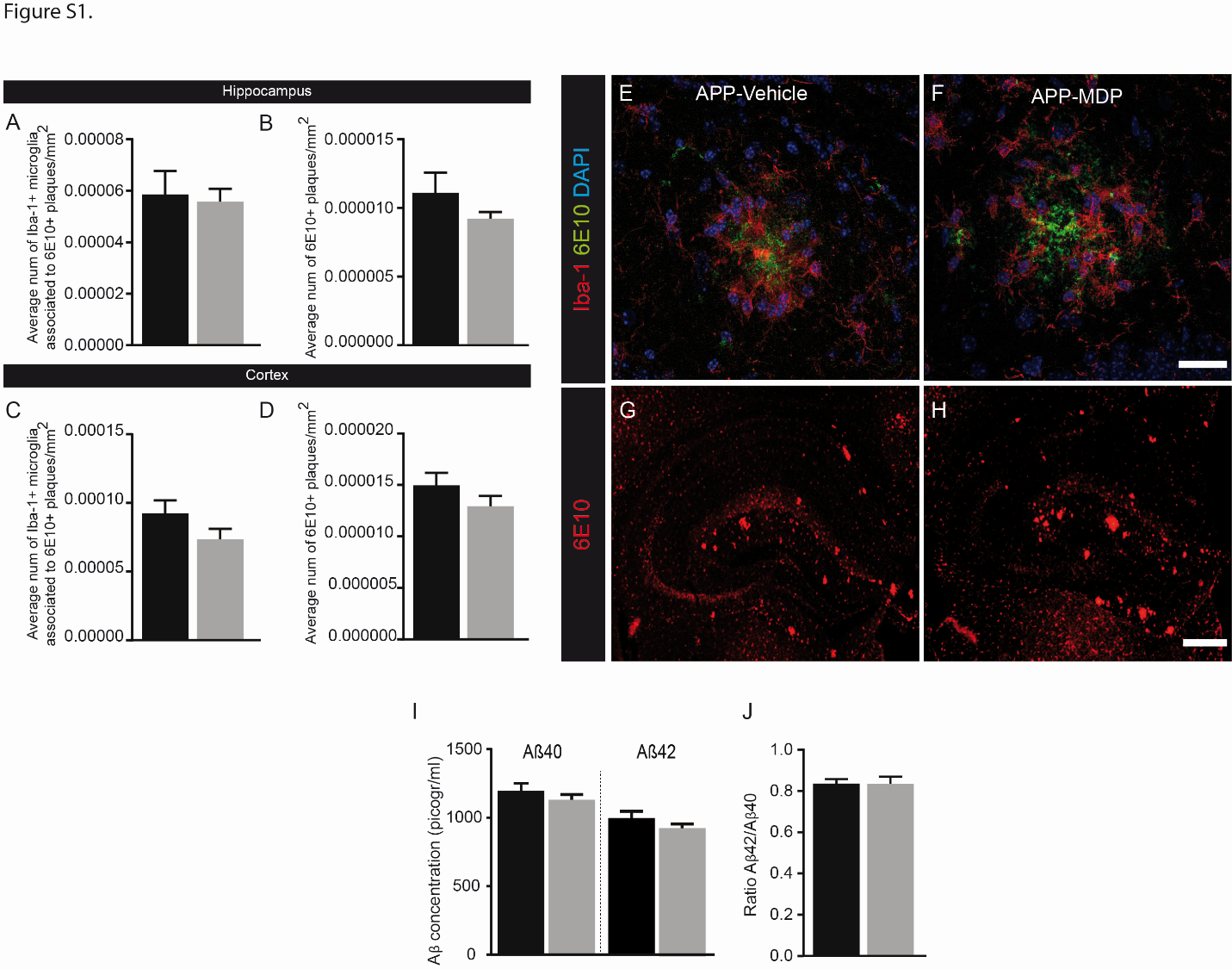
**

(A) and (B) The average number of Iba1+ associated to 6E10+ plaques, and the average number of 6E10+ plaques respectively to the hippocampus area (um2) of APP mice treated with vehicle and MDP (n=10 mice per group, and 4-6 sections/mouse).

(C) and (D) The average number of Iba1+ associated to 6E10+ plaques, and the average number of 6E10+ plaques respectively to cortex area (um2) of APP mice treated with vehicle and MDP(n=10 mice per group, and 4-6 sections/mouse).

(E) and (F) Representative iba1 (red), 6E10 (green), and DAPI (blue)-immunoreactivity in the hippocampus of APP mice treated with vehicle (left) and MDP (right) (Scale bar, 20μm).

(G) and (H) Representative 6E10 (red)-immunoreactivity in the hippocampus of APP mice treated with vehicle (left) and MDP (right) (Scale bar, 100μm).

(I) Aβ40 and Aβ42 concentrations (picogram/ml) in the cortex and hippocampus of APP mice treated with vehicle and MDP were quantified by ELISA (n=10 mice per group).

(J) Aβ40 and Aβ42 ratios in the cortex and hippocampus of APP mice treated with vehicle and MDP, which were quantified by ELISA (n=10 mice per group).

# B. Flow cytometry

**B1. Flow cytometry gating strategy**


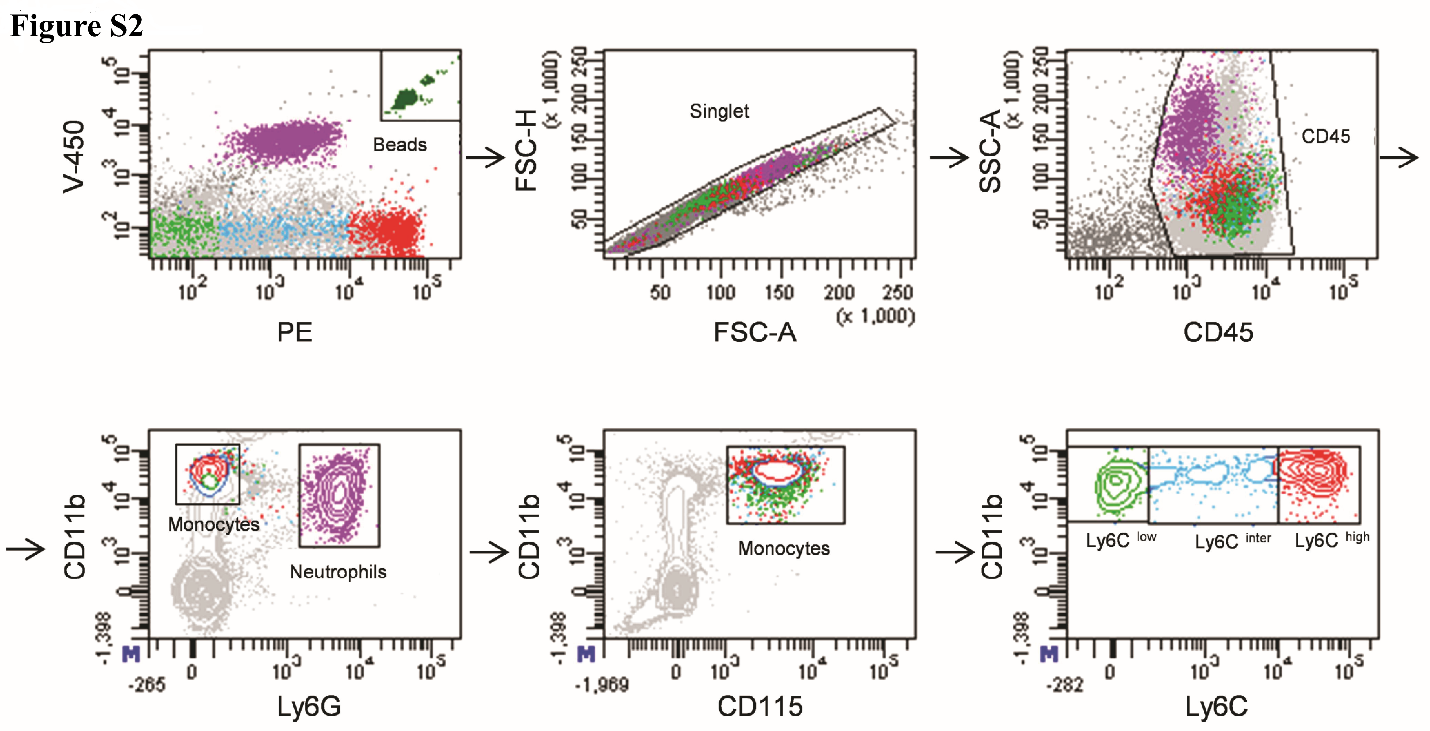


Figure S2. Flow cytometry gating strategy of monocytes and monocyte subsets.

Representative gating strategy for CD11b^+^ CD115^+^ monocyte and Ly6C monocyte subsets for all experiments and mouse models. To identify absolute counting of cell populations, 123count eBeads™ are gated. Bead population excluded, and doublet discrimination is performed with a singlet gate (FSC-H/FSC-A dot blot). Dead/live analysis was performed (not shown here). Next, CD45^+^/CD11b^+^/Ly6G^+^ cells were considered as neutrophils. Neutrophil cell population gated out. Next, monocytes were identified with CD45, CD11b and CD115 expression. Monocyte subsets were further subdivided into three populations based on the expression of Ly6C: Ly6C^high^, Ly6C^int^ and Ly6C^low^ correspond respectively to inflammatory, intermediate and patrolling monocytes. Pictures were adopted from [1].

**B.2 Flow cytometry protocol**

Briefly, 50 µl of total blood was diluted with 35 µl of DPBS without Ca^2+^ or Mg^2+^ and incubated 15 min on ice with purified rat anti–mouse CD16/CD32 antibody (Mouse BD Fc Block; BD). Cells were then labeled at 4°C during 40 min with the following rat anti–mouse antibodies: V500-conjugated anti-CD45 antibody (1/100, BD BioScience), AF700-conjugated anti-CD11b antibody (1/100, eBioscience), APC (allophycocyanin)-conjugated anti-CD115 antibody (1/100, eBioscience), V450-conjugated anti-Ly6C antibody (1/100, BD BioScience) and PE-conjugated anti-Ly6G antibody (1/100, eBioscience), FITC-conjugated anti-CD19, and Live/Dead Fixable Blue Dead Cell Stain (Invitrogen, Paisley, UK). Next, red blood cells were lysed with 1.5 ml of 1X Pharm Lyse buffer (BD BioScience) during 20 min at room temperature, and the remaining leukocytes were washed and resuspended with DPBS without Ca^2+^ and Mg^2+^. More information about the procedure can be found at[2]

1. Lessard A-J, LeBel M, Egarnes B, Préfontaine P, Thériault P, Droit A, Brunet A, Rivest S, Gosselin J: **Triggering of NOD2 receptor converts inflammatory Ly6Chigh into Ly6Clow monocytes with patrolling properties.** *Cell reports* 2017, **20:**1830-1843.

2. Thériault P, ElAli A, Rivest S: **High fat diet exacerbates Alzheimer's disease-related pathology in APPswe/PS1 mice.** *Oncotarget* 2016, **7:**67808.
